# Prevalence, Correlates, and Prognostic Significance of In-Hospital Transthoracic Echocardiography Use in Stable Acute Myocardial Infarction

**DOI:** 10.3390/jcdd13070322

**Published:** 2026-07-10

**Authors:** Alon Shechter, Arthur Shiyovich, Robert J. Siegel, Olga Morelli, Harel Gilutz, Ygal Plakht

**Affiliations:** 1 Department of Cardiology, Rabin Medical Center, Petach Tikva 4910000, Israelolgamorelli55@gmail.com (O.M.); 2 Gray Faculty of Medical and Health Sciences, Tel Aviv University, Tel Aviv 6997801, Israel; 3 Division of Cardiovascular Medicine, Department of Medicine, Brigham and Women’s Hospital, Boston, MA 02115, USA; arthur.shiyovich@gmail.com; 4 Cardiovascular Division, Department of Medicine, Harvard Medical School, Boston, MA 02115, USA; 5 Department of Cardiology, Smidt Heart Institute, Cedars-Sinai Medical Center, Los Angeles, CA 90048, USA; echorjs@gmail.com; 6 David Geffen School of Medicine, University of California Los Angeles, Los Angeles, CA 90095, USA; 7 Goldman Medical School, Faculty of Health Sciences, Ben-Gurion University of the Negev, Beer Sheva 8410501, Israel; gilutz@bgu.ac.il; 8 Department of Nursing, Recanati School for Community Health Professions, Faculty of Health Sciences, Ben-Gurion University of the Negev, Beer Sheva 8410501, Israel; 9 Department of Emergency Medicine, Soroka University Medical Center, Beer Sheva 8410101, Israel

**Keywords:** myocardial infarction, prognosis, survival, transthoracic echocardiography

## Abstract

Little is known regarding in-hospital transthoracic echocardiography (TTE) utilization and its prognostic implications among stable patients with acute myocardial infarction (AMI). We aimed to explore patient and disease characteristics, treatment strategies, and mid-term outcome following uncomplicated AMI according to TTE use during the hospitalization phase. A single-center, retrospective analysis was conducted that included consecutive adult individuals admitted for AMI who did not develop cardiogenic shock and who survived the index hospitalization. Stratified by in-hospital TTE administration status, the cohort was evaluated for all-cause mortality at 1-year post-discharge. Overall, 15,971 subjects (mean age 66 ± 14 years, 69.8% males, 46.1% with ST-elevation myocardial infarction) were analyzed, of whom 12,610 (79.0%) underwent TTE. TTE use correlated with younger age, fewer comorbidities, greater odds of invasive revascularization and intensive coronary care unit management, and lengthier hospital stay. Ultimately, it was associated with a lower rate, cumulative incidence, and—independent of accompanying prognostic markers—risk of all-cause mortality (n = 1032/12,619, 8.2% vs. n = 804/3361, 23.9%, *p* < 0.001; Log-Rank *p* < 0.001; adjusted hazard ratio 0.75, 95% confidence interval 0.67–0.83, *p* < 0.001). Similar results were observed within a 6270-patient, 1-1 propensity score-matched sub-cohort. To conclude, in our experience, in-hospital TTE administered for stable AMI patients was associated with improved mid-term survival. Further research is needed to re-evaluate the present-day recommendation’s Level of Evidence C for its routine use.

## 1. Introduction

A simple, readily available, bedside non-invasive imaging modality, transthoracic echocardiography (TTE), is potentially advantageous in subjects with acute coronary syndromes, as it allows for the appreciation of cardiac structure and function as well as hemodynamic status, thus assisting treating teams in recognizing therapeutic targets, formulating management strategies and, ultimately, enhancing downstream patient course [1]. Nevertheless, currently there are only limited data to support the theoretical prognostic benefit associated with routinely performing TTE in stable acute myocardial infarction (AMI) patients during hospitalization, as reflected by a Level of Evidence C recommendation for such practice in the most recent European [2] and American [3] guidelines. To address this knowledge gap, and specifically test the hypothesis linking TTE administration with improved survival following uncomplicated AMI, we analyzed a large, contemporary database according to TTE use during the index admission.

## 2. Materials and Methods

### 2.1. Study Population and Outcome

Our study represents a sub-analysis of the previously described [4,5] Soroka Acute Myocardial Infarction (SAMI) registry. Included in the present, retrospective study were adult (i.e., ≥18-year-old) Israeli citizens admitted for AMI at Soroka University Medical Center, Israel, between 2002 and 2017 who did not develop cardiogenic shock and who survived the acute hospitalization phase. For subjects with multiple hospitalizations, we considered only the initial one. The study outcome was all-cause mortality during the first year after discharge.

Conforming to the Declaration of Helsinki, the project was approved by Soroka’s Institutional Review Board (approval number SOR-0319-16), and the need for informed consent was waived.

### 2.2. Data Collection and Definitions

Clinical data were retrieved from a web-based medical chart, in which baseline comorbidities were identified by International Classification of Diseases, Ninth Revision, Clinical Modification (ICD-9-CM) [6] codes, as documented in real-time by the treating physicians and according to accepted diagnostic criteria. Deaths were confirmed using the Israeli Ministry of the Interior Population Registry.

AMI diagnosis was based upon the constellation of ischemic signs and/or symptoms coupled with an abrupt rise and fall in cardiac biomarker levels consistent with acute myocardial injury, as dictated by the Universal Definition of Myocardial Infarction at the time [7]. Diagnosis of obstructive coronary artery disease required angiographic evidence of a ≥70% vessel diameter narrowing.

Echocardiographic diagnoses aligned with the American Society of Echocardiography guidelines. Accordingly, severe left ventricular dysfunction was defined by an ejection fraction of <30%, and pulmonary hypertension as an estimated pulmonary arterial systolic pressure of ≥37 mmHg.

### 2.3. Statistical Analysis

Both the entire cohort and a propensity score (PS)-matched sub-cohort were analyzed according to the administration of in-hospital TTE. Within the latter, pairing was dictated by the probability of undergoing TTE, using a nearest neighbor, 1-to-1 matching method, and a tolerance of ≤0.2. Included in the binary logistic regression model used for predicting this probability were baseline/presenting characteristics of known or perceived prognostic significance, all selected prior to the execution of descriptive analyses: increasing age, male sex, atherosclerotic risk factors, chronic obstructive pulmonary disease, chronic kidney disease, pre-existent chronic coronary syndrome, clinical heart failure, right heart failure, atrial fibrillation/flutter, and more recent year of event. Between-group balance for each of PS variable was assessed using absolute standardized differences, as depicted by a Love plot (Figure S1). In addition, discriminative ability of the score was checked using Harrell’s C-statistic, which proved acceptable (0.72, 95% CI 0.71–0.73, *p* < 0.001).

At all stages, variables were reported as frequencies and percentages or means and standard deviations and compared using Pearson’s Chi-Square or Student’s *t* tests, as appropriate.

The time-dependent probability and cumulative incidence of mortality as a function of TTE performance were assessed by the Kaplan–Meier method and compared using the Log-Rank test. Independent associations with the risk for all-cause death were evaluated by a Cox proportional hazard multivariable analysis and expressed using each’s unadjusted hazard ratio (HR)/adjusted HR (AdjHR) and the latter’s 95% confidence interval (CI). The model incorporated covariates exhibiting a *p*-value of <0.1 on the univariable stage. The proportional hazards assumption was evaluated qualitatively by checking for parallel orientation of the log-minus-log survival plots for all covariates. Model fit was assessed using the −2 log likelihood and likelihood ratio test.

Statistical significance required a two-sided *p*-value of <0.05. All analyses were performed using Statistical Package for the Social Sciences (SPSS), version 31 (IBM Corporation, Armonk, NY, USA).

## 3. Results

### 3.1. Baseline Characteristics of the Study Population

**Figure 1:**
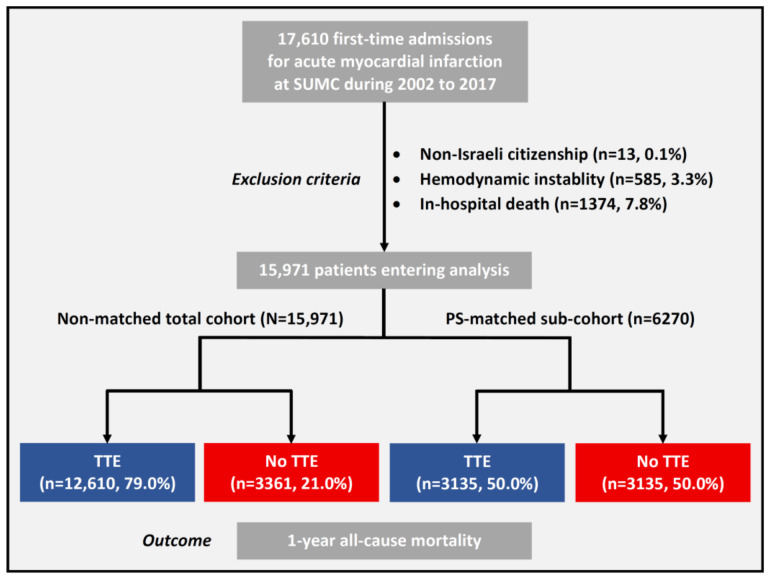
Study flow chart. SUMC = Soroka University Medical Center; TTE = transthoracic echocardiography.

A total of 15,971 individuals qualified for analysis (Figure 1). Of them, 12,610 (79.0%) underwent TTE during their hospital stay. Notably, TTE utilization rose during the study recruitment period, from 74.2% in 2002 to 86.6% in 2017 (*p* for trend <0.001).

Compared to patients who were not scanned, those within the TTE group were recruited to the study at a later calendar year and were younger and more likely to be males of a non-Jewish minority background (Table 1). They also exhibited an overall higher burden of atherosclerotic cardiovascular risk factors but fewer comorbidities including overt heart failure. Echocardiograms demonstrated severe left ventricular dysfunction in 1316 (8.2%) out of the 12,610 cases referred for TTE, right ventricular dysfunction in 971 (7.7%), moderate and above mitral regurgitation in 670 (4.2%), moderate and above tricuspid regurgitation in 426 (2.7%), and pulmonary hypertension in 913 (5.7%).

### 3.2. Acute Event Aspects

Subjects who underwent TTE presented more commonly with ST elevation myocardial infarction (STEMI) (n = 6356, 50.4% vs. n = 1005, 29.9%, *p* < 0.001) and with a similarly low rate (0.3%) of cardiac arrest compared to those who did not undergo TTE (Table 2). The former were more likely to be assessed angiographically and to obtain definitive revascularization, leading to more intensive coronary care unit admissions/transfers and longer hospital stays. Also, their hospitalization courses were characterized by more frequent arrhythmic sequelae and fewer bleeding complications and sepsis. Specifically among patients who underwent both TTE and invasive revascularization and for whom data regarding respective timings were available (n = 10,356), TTE preceded revascularization by more than a day in 1129 (10.9%) cases, took place within a day of revascularization in 7238 (70.0%), or was performed more than a day after revascularization in 1989 (19.2%).

### 3.3. Outcome

**Figure 2:**
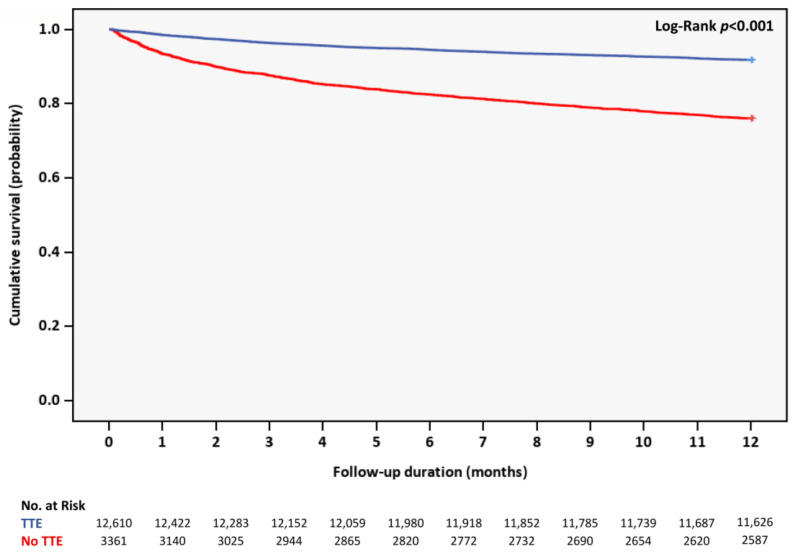
Cumulative survival according to transthoracic echocardiography use. TTE = transthoracic echocardiography.

By 1 year after discharge, 1836 (11.5%) patients died. TTE performance was associated with a lower rate, cumulative incidence, and—independent of accompanying prognostic markers at baseline and during hospitalization—risk of all-cause mortality (n = 1032/12,619, 8.2% vs. n = 804/3361, 23.9%, *p* < 0.001; Log-Rank *p* < 0.001; unadjusted HR 0.31, 95% CI 0.28–0.34, *p* < 0.001; AdjHR 0.75, 95% CI 0.67–0.83, *p* < 0.001) (Figure 2 and Table 3).

Of note, within the TTE/invasive revascularization subgroup, death rate was highest among patients scanned after revascularization (n = 187/1989, 9.4%), intermediate among those scanned before revascularization (n = 48/1129, 4.3%), and lowest among those scanned surrounding revascularization (n = 284/7238, 3.9%) (*p* < 0.001).

Per exploratory analysis, the significant association between TTE performance and reduced mortality risk was confined to patients presenting with non-ST elevation MI (NSTEMI) (AdjHR 0.71, 95% CI 0.63–0.80, *p* < 0.001) and those managed non-invasively (AdjHR 0.72, 95% CI 0.64–0.81, *p* < 0.001), as opposed to patients with STEMI (AdjHR 0.92, 95% CI 0.74–1.13, *p* = 0.417) and those referred to invasive revascularization (AdjHR 0.88, 95% CI 0.69–1.13, *p* = 0.328).

### 3.4. Propensity Score Matching Analysis

**Figure 3:**
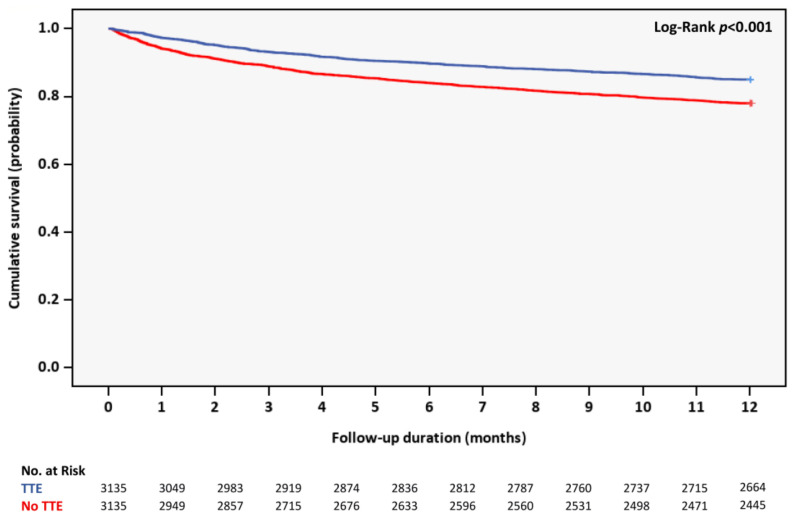
Cumulative survival according to transthoracic echocardiography use within the propensity score-matched sub-cohort. TTE = transthoracic echocardiography.

Within a 6270-case PS-matched sub-cohort, composed of 3135 patients who underwent TTE and 3135 matched controls who did not, most differences in baseline characteristics and acute event aspects became neutralized (Table 4 and Table 5). Still, ST elevation, angiographic assessment, invasive revascularization, and intensive care unit management were more common in the TTE group. Similar to the total unmatched cohort, patients who underwent TTE experienced fewer, more distant death events (n = 471, 15.0% vs. n = 690, 22.0%, *p* < 0.001; Log-Rank *p* < 0.001), and TTE utilization conferred a lower death risk (unadjusted HR 0.65, 95% CI 0.58–0.73, *p* < 0.001; AdjHR 0.77, 95% CI 0.68–0.88, *p* < 0.001) (Figure 3 and Table 6). Likewise, the prognostic significance of TTE administration was only observed among NSTEMI cases (AdjHR 0.75, 95% CI 0.65–0.86, *p* < 0.001) and non-invasively managed patients (AdjHR 0.75, 95% CI 0.65–0.86, *p* < 0.001), as opposed to STEMI scenarios (AdjHR 0.89, 95% CI 0.68–1.16, *p* = 0.369) and invasively treated subjects (AdjHR 0.90, 95% CI 0.67–1.21, *p* = 0.492).

## 4. Discussion

In this single-center, retrospective study, we examined the utilization of in-hospital TTE among 15,971 real-world patients admitted for non-fatal, stable AMI over a period of 15 years. Its results revealed the following: 1. TTE was performed more frequently in later years and in almost 80% of subjects overall. 2. Compared to patients who did not undergo TTE during the index hospitalization, those who did presented at a younger age and with fewer comorbidities, were more often managed invasively and within the intensive coronary care unit, and exhibited a lengthier hospital stay. 3. TTE performance was associated with improved 1-year survival, independently conferring a mortality risk that was 1.3 times lower than that observed within the no-TTE group.

The current literature concerning the prevalence, correlates, and prognostic significance of in-hospital TTE administration in AMI is limited. In the largest report prior to ours, covering almost 100,000 admissions across nearly 400 centers in the United States, TTE was performed in 74% of cases and a more liberal use of TTE correlated with higher costs and longer hospitalizations without affecting inpatient mortality nor 3-month readmissions [8]. Importantly, the authors relied on an administrative, billing code-focused database that spanned merely one year of practice (2014) and which included a highly heterogenous population treated in centers of varying capabilities (with some not offering percutaneous coronary intervention services at all). Moreover, the study’s analyses revolved around inter-center comparisons rather than patient-level, actual administration of TTE per se, all while considering stable/unstable patients and first-time/repeat hospitalizations as one. An earlier study (2001–2011) examining the use of echocardiography in various medical conditions identified enhanced in-hospital survival among AMI patients who underwent TTE [9]. However, echocardiography was performed in only 7% of cases and no adjustment was made for deaths occurring prior to the performance of TTE. Bearing in mind these previous works’ setbacks, we designed the present study which focused on mid-term survival post-AMI, presumably providing a more accurate reflection of TTE utilization.

Our observations have two clinically relevant implications. The first is that performing TTE during hospitalization for AMI was linked to better prognosis following discharge. It could be that earlier detection of actionable conditions known to affect survival, such as systolic dysfunction, diastolic impairment, valvular disorders, and structural/thrombotic complications allowed for a more comprehensive management (that is, beyond mere revascularization) within the TTE group that in turn led to improved outcomes. Consistent with this assumption were: a. the lower death rates reported among revascularized patients who underwent TTE at an earlier stage; and b. the confinement of the reduced death hazard associated with TTE use to patients presenting with NSTEMI or who were managed non-invasively, both of whom are traditionally less exposed to prognostically meaningful interventions and may thus derive added benefit from TTE. Alternatively, the administration of TTE may have simply served as a surrogate of stricter guideline-directed therapy adherence and hence higher standards of care. This hypothesis fits well with the observation linking TTE performance with greater utilization of invasive revascularization and intensive care environment and more advanced year of event, although the latter did not alter the independent prognostic value of TTE use (and, furthermore, intensive care unit stay and event year were not associated with survival on their own). As our registry lacked information regarding full TTE reports, their impact on management strategy, death causes, and clinical pathways leading to TTE use in the first place, the exact mechanism(s) underlying our findings remain speculative. Accordingly, and as much as the study’s extensive multivariable analyses implied the independent predictive role of TTE use, further, preferentially prospective validation is needed.

The second message offered by our study is that TTE was relatively underutilized during the hospitalization phase surrounding AMI diagnosis, much in the same manner reported by prior publications [10]. In light of the observed intergroup differences in patient characteristics and revascularization approach, it could be that TTE was perceived as lower-yield in those subjects who were deemed too high risk or unfit for interventional therapies a priori. Technical obstacles, too, may have contributed to the low usage of TTE in persons presenting with numerous comorbidities, in whom hand-held echocardiography could constitute a similarly accurate and more feasible alternative [11]. Once again, we were unable to identify causality, thus we are leaving it for future studies to better define barriers for the implementation of TTE in this setting.

### Limitations

First, our study stemmed from a single-center, retrospective analysis that did not employ randomization nor external adjudication, potentially leading to selection bias and hampering the generalizability of the results. However, we relied on a large cohort that represented a population of close to 1 million and whose baseline characteristics resembled those of prior registries. Also, randomized-controlled trials of TTE utilization among stable AMI patients are hardly pragmatic, arguably making real-world exploration as our the next best alternative. Secondly, and again reflecting the study’s retrospective design, statistical precision and associated clinical relevance of our findings may be questionable. Yet, based on the sample size, exposure (i.e., TTE use) distribution, event rate, and Cox model utilized, and assuming a two-sided α of 0.05 and 80% power, we estimated the minimum detectable effect size as HR for all-cause mortality of approximately 0.90, a figure which is generally considered clinically meaningful in cardiovascular outcome research, particularly in settings where imaging may influence downstream decision-making rather than exert direct therapeutic effects. Thirdly, aiming to focus on patients for whom current practice guidelines provide expert opinion-based recommendation only, and in view of the study’s objective to explore the 1-year trajectory of these individuals, we deliberately excluded in-hospital fatalities, hence introducing survivor bias. This could also mitigate the possibility of immortal time bias and in essence led to a modest 7.8% exclusion rate (Figure 1). Fourthly, the protracted timeframe of enrollment may have led to inconsistencies in medical definitions and treatment approaches, which also could have affected the interpretation of the findings. Nevertheless, both study groups were exposed to similarly evolving diagnostic criteria and practices, and admission year did not possess an independent prognostic capacity nor did it undermine the significant association demonstrated between TTE performance and 1-year mortality. Fifthly, in addition to death causes, we were also unaware of the exact TTE scope (complete vs. focused), and—in some patients—timing, as well as MI types (e.g., type 2) and extent (e.g., Killip class, serum biomarker levels), medical therapies other than blood transfusion, and reason for re-admissions. Consequently, we could not comment on the pathophysiologic basis accounting for our observations nor rule out confounding by non-coronary-related phenomena. Lastly, and in view of the evaluation of multiple covariates by a Cox regression model, chance findings could not be entirely excluded, necessitating cautious interpretation which takes into consideration effect size (and the possibility of smaller effect sizes than the one estimated above), confidence intervals, and their clinical plausibility.

## 5. Conclusions

In our large, single-center experience, in-hospital TTE was utilized in close to 80% of subjects with stable, non-fatal AMI and was associated with a lower-risk patient profile, a higher rate of invasive coronary interventions and intensive care management, and a more favorable 1-year survival. These findings call for further assessment of the routine use of TTE in such scenarios.

## Figures and Tables

**Table 1:** Baseline clinical characteristics.

	Total Cohort (N = 15,971)	Transthoracic Echocardiography Use		*p*-Value
		Yes (n = 12,610)	No (n = 3361)	
**Demographic details**				
Age				**<0.001**
Continuous (years)	66 ± 14	64 ± 13	72 ± 14	
<65 years	7926 (49.6)	6959 (55.2)	967 (28.8)	
65–74 years	3623 (22.7)	2836 (22.5)	787 (23.4)	
≥75 years	4422 (27.7)	2815 (22.3)	1607 (47.8)	
Sex male	11,152 (69.8)	9165 (72.7)	1987 (59.1)	**<0.001**
Non-Jewish minority	2651 (16.6)	2213 (17.5)	438 (13.0)	**<0.001**
**Atherosclerotic** **cardiovascular risk factors**				
Diabetes mellitus	6444 (40.3)	4912 (39.0)	1532 (45.6)	**<0.001**
Dyslipidemia	12,975 (81.2)	10,556 (83.7)	2419 (72.0)	**<0.001**
Hypertension	8280 (51.8)	6438 (51.1)	1842 (54.8)	**<0.001**
Obesity	3579 (22.4)	3033 (24.1)	546 (16.2)	**<0.001**
Smoking history	6743 (42.2)	5879 (46.6)	864 (25.7)	**<0.001**
Family history of ischemic heart disease	1494 (9.4)	1360 (10.8)	134 (4.0)	**<0.001**
**Cardiovascular** **comorbidities**				
Chronic coronary syndrome	12,496 (78.2)	10,473 (83.1)	2023 (60.2)	**<0.001**
History of myocardial infarction	1786 (11.2)	1320 (10.5)	466 (13.9)	**<0.001**
Prior revascularization				
Percutaneous coronary intervention	1907 (11.9)	1493 (11.8)	414 (12.3)	0.448
Coronary artery bypass grafting	1243 (7.8)	889 (7.0)	354 (10.5)	**<0.001**
Peripheral arterial disease	1799 (11.3)	1337 (10.6)	462 (13.7)	0.266
Atrial fibrillation/flutter	2447 (15.3)	1738 (13.8)	709 (21.1)	**<0.001**
Atrioventricular block	592 (3.7)	470 (3.7)	122 (3.6)	0.791
Clinical heart failure	2556 (16.0)	1870 (14.8)	686 (20.4)	**<0.001**
**Non-cardiovascular** **comorbidities**				
Chronic obstructive pulmonary disease	1232 (7.7)	880 (7.0)	352 (10.5)	**<0.001**
Stage ≥III chronic kidney disease	5157 (32.3)	3749 (29.7)	1408 (41.9)	**<0.001**
Anemia	8498 (53.2)	6437 (51.0)	2061 (61.3)	**<0.001**
Neurological disorders	2567 (16.1)	1710 (13.6)	857 (25.5)	**<0.001**
Malignancy	612 (3.8)	422 (3.3)	190 (5.7)	**<0.001**
Substance use disorder	340 (2.1)	284 (2.3)	56 (1.7)	**0.036**
Psychotic disorders	258 (1.6)	177 (1.4)	81 (2.4)	**<0.001**

Data are presented as number (percent) or mean ± standard deviation. Figures in bold denote statistical significance.

**Table 2:** Acute event aspects.

	Total Cohort (N = 15,971)	Transthoracic Echocardiography Use		*p*-Value
		Yes (n = 12,610)	No (n = 3361)	
**Clinical presentation**				
ST elevation myocardial infarction	7361 (46.1)	6356 (50.4)	1005 (29.9)	**<0.001**
Cardiac arrest	52 (0.3)	43 (0.3)	9 (0.3)	0.508
Right heart failure	1251 (7.8)	971 (7.7)	280 (8.3)	0.227
**Angiographic parameters**				
Angiogram performed	11,038 (69.1)	10,356 (82.1)	682 (20.3)	**<0.001**
Vessels significantly involved				**<0.001**
0	507 (4.6)	446 (4.3)	61 (8.9)	
1	3076 (27.9)	2913 (28.1)	163 (23.9)	
2	3092 (28.0)	2913 (28.1)	179 (26.2)	
3/Left main	4363 (39.5)	4084 (39.4)	279 (40.9)	
**Hospital course**				
Revascularization approach				**<0.001**
None/ conservative treatment	4207 (26.3)	2087 (16.6)	2120 (63.1)	
Percutaneous coronary intervention	9720 (60.9)	8753 (69.4)	967 (28.8)	
Coronary artery bypass grafting	2044 (12.8)	1770 (14.0)	274 (8.2)	
Intensive coronary care unit stay	11,029 (69.1)	10,119 (80.2)	910 (27.1)	**<0.001**
Ventricular tachycardia	378 (2.4)	332 (2.6)	46 (1.4)	**<0.001**
Any form of pacing	284 (1.8)	269 (2.1)	15 (0.4)	**<0.001**
Mechanical ventilation	548 (3.4)	438 (3.5)	110 (3.3)	0.570
Gastrointestinal bleeding	320 (2.0)	242 (1.9)	78 (2.3)	0.140
Blood transfusion	1887 (11.8)	1449 (11.5)	438 (13.0)	**0.014**
Sepsis	124 (0.8)	85 (0.7)	39 (1.2)	**0.004**
Hospitalization length				
Continuous (days)	10.0 ± 9.0	10.3 ± 9.2	8.8 ± 8.1	**<0.001**
≥7 days	7538 (47.2)	6207 (49.2)	1331 (39.6)	**<0.001**

Data are presented as number (percent) or mean ± standard deviation. Figures in bold denote statistical significance.

**Table 3:** Multivariable Cox proportional hazard model for the outcome of all-cause mortality at 1 year.

	Univariable		Multivariable	
	HR (95% CI)	*p*-Value	AdjHR (95% CI)	*p*-Value
**Year of admission** **(continuous, per 1-year increase)**	0.97 (0.96–0.98)	**<0.001**	1.00 (0.99–1.02)	0.456
**Demographic details**				
Age ≥ 65 vs. <65 years	4.42 (3.78–5.18)	**<0.001**	2.26 (1.91–2.66)	**<0.001**
Sex male	0.52 (0.47–0.57)	**<0.001**	0.92 (0.83–1.02)	0.102
**Atherosclerotic** **cardiovascular risk factors**				
Dyslipidemia	0.46 (0.41–0.50)	**<0.001**	0.75 (0.68–0.83)	**<0.001**
Obesity	0.49 (0.42–0.56)	**<0.001**	0.77 (0.67–0.88)	**<0.001**
Diabetes mellitus	1.51 (1.42–1.70)	**<0.001**	1.18 (1.07–1.30)	**<0.001**
**Cardiovascular** **comorbidities**				
Chronic coronary syndrome	0.30 (0.27–0.33)	**<0.001**	0.81 (0.73–0.91)	**<0.001**
Peripheral arterial disease	2.35 (2.10–2.62)	**<0.001**	1.49 (1.32–1.67)	**<0.001**
Atrial fibrillation/flutter	2.62 (2.38–2.90)	**<0.001**	1.28 (1.16–1.42)	**<0.001**
Clinical heart failure	2.75 (2.49–3.03)	**<0.001**	1.31 (1.18–1.46)	**<0.001**
**Non-cardiovascular** **comorbidities**				
Chronic obstructive pulmonary disease	2.55 (2.25–2.88)	**<0.001**	1.59 (1.40–1.81)	**<0.001**
Stage ≥ III chronic kidney disease	3.36 (3.06–3.69)	**<0.001**	1.42 (1.28–1.58)	**<0.001**
Anemia	3.13 (2.81–3.49)	**<0.001**	1.56 (1.39–1.75)	**<0.001**
Neurological disorders	3.22 (2.93–3.54)	**<0.001**	1.57 (1.42–1.74)	**<0.001**
Malignancy	3.88 (3.37–4.47)	**<0.001**	2.32 (2.00–2.68)	**<0.001**
Substance use disorder	2.19 (1.69–2.83)	**<0.001**	1.83 (1.37–2.46)	**<0.001**
Psychotic disorders	2.55 (2.25–2.88)	**<0.001**	1.46 (1.12–1.88)	**0.004**
**Clinical presentation**				
ST elevation (vs. non-ST elevation) myocardial infarction	0.46 (0.42–0.51)	**<0.001**	1.01 (0.90–1.13)	0.880
Right heart failure	2.64 (2.34–2.98)	**<0.001**	1.39 (1.23–1.58)	**<0.001**
**Hospital course**				
Invasive revascularization vs. conservative approach	0.15 (0.14–0.17)	**<0.001**	0.45 (0.39–0.52)	**<0.001**
Mechanical ventilation	2.50 (2.10–2.98)	**<0.001**	1.34 (1.12–1.61)	**0.001**
**Transthoracic** **echocardiography use**	0.31 (0.28–0.34)	**<0.001**	0.75 (0.67–0.83)	**<0.001**

Figures in bold denote statistical significance. AdjHR = adjusted hazard ratio; CI = confidence interval; HR = hazard ratio.

**Table 4:** Baseline clinical characteristics of the propensity score-matched sub-cohort.

	Propensity Score-Matched Sub-Cohort (N = 6270)	Transthoracic Echocardiography Use		*p*-Value
		Yes (n = 3135)	No (n = 3135)	
**Demographic details**				
Age				
Continuous (years)	71.42 ± 13.04	71.4 ± 12.59	71.41 ± 13.48	0.938
<65 years	1953 (31.1)	986 (31.5)	967 (30.8)	0.711
65–74 years	1533 (24.4)	753 (24.0)	780 (24.9)	
≥75 years	2784 (44.4)	1396 (44.5)	1388 (44.3)	
Sex male	3833 (61.1)	1921 (61.3)	1912 (61.0)	0.816
Non-Jewish minority	455 (7.3)	31 (1.0)	424 (13.5)	**<0.001**
**Atherosclerotic** **cardiovascular risk factors**				
Diabetes mellitus	2808 (44.8)	1379 (44.0)	1429 (45.6)	0.204
Dyslipidemia	4736 (75.5)	2380 (75.9)	2356 (75.2)	0.481
Hypertension	3448 (55.0)	1720 (54.9)	1728 (55.1)	0.839
Obesity	1098 (17.5)	557 (17.8)	541 (17.3)	0.595
Smoking history	1696 (27.0)	837 (26.7)	859 (27.4)	0.532
Family history of ischemic heart disease	323 (5.2)	189 (6.0)	134 (4.3)	**0.002**
**Cardiovascular** **comorbidities**				
Chronic coronary syndrome	4020 (64.1)	2000 (63.8)	2020 (64.4)	0.598
History of myocardial infarction	732 (11.7)	270 (8.6)	462 (14.7)	**<0.001**
Prior revascularization				
Percutaneous coronary intervention	716 (11.4)	303 (9.7)	413 (13.2)	**<0.001**
Coronary artery bypass grafting	588 (9.4)	235 (7.5)	353 (11.3)	**<0.001**
Peripheral arterial disease	801 (12.8)	369 (11.8)	432 (13.8)	**0.017**
Atrial fibrillation/flutter	1304 (20.8)	658 (21.0)	646 (20.6)	0.709
Atrioventricular block	281 (4.5)	163 (5.2)	118 (3.8)	**0.006**
Clinical heart failure	1253 (20.0)	631 (20.1)	622 (19.8)	0.776
**Non-cardiovascular** **comorbidities**				
Chronic obstructive pulmonary disease	589 (9.4)	263 (8.4)	326 (10.4)	**0.006**
Stage ≥ III chronic kidney disease	2556 (40.8)	1266 (40.4)	1290 (41.1)	0.537
Anemia	3741 (59.7)	1848 (58.9)	1893 (60.4)	0.247
Neurological disorders	1432 (22.8)	716 (22.8)	716 (22.8)	1.000
Malignancy	320 (5.1)	149 (4.8)	171 (5.5)	0.207
Substance use disorder	108 (1.7)	54 (1.7)	54 (1.7)	1.000
Psychotic disorders	149 (2.4)	70 (2.2)	79 (2.5)	0.456

Data are presented as number (percent) or mean ± standard deviation. Figures in bold denote statistical significance.

**Table 5:** Acute event aspects within the propensity score-matched sub-cohort.

	Propensity Score-Matched Sub-Cohort (N = 6270)	Transthoracic Echocardiography Use		*p*-Value
		Yes (n = 3135)	No (n = 3135)	
**Clinical presentation**				
ST elevation myocardial infarction	2327 (37.1)	1363 (43.5)	964 (30.7)	**<0.001**
Cardiac arrest	17 (0.3)	10 (0.3)	7 (0.2)	0.466
Right heart failure	569 (9.1)	291 (9.3)	278 (8.9)	0.568
**Angiographic parameters**				
Angiogram performed	2723 (43.4)	2042 (65.1)	681 (21.7)	**<0.001**
Vessels significantly involved				0.198
0	197 (7.2)	136 (6.7)	61 (9.0)	
1	640 (23.5)	477 (23.4)	163 (23.9)	
2	758 (27.8)	579 (28.4)	179 (26.3)	
3/Left main	1128 (41.4)	850 (41.6)	278 (40.8)	
**Hospital course**				
Revascularization approach				**<0.001**
None/ conservative treatment	2948 (47.0)	1052 (33.6)	1896 (60.5)	
Percutaneous coronary intervention	2699 (43.0)	1734 (55.3)	965 (30.8)	
Coronary artery bypass grafting	623 (9.9)	349 (11.1)	274 (8.7)	
Intensive coronary care unit stay	2969 (47.4)	2062 (65.8)	907 (28.9)	**<0.001**
Ventricular tachycardia	107 (1.7)	61 (1.9)	46 (1.5)	0.144
Any form of pacing	103 (1.6)	88 (2.8)	15 (0.5)	**<0.001**
Mechanical ventilation	227 (3.6)	124 (4.0)	103 (3.3)	0.156
Gastrointestinal bleeding	149 (2.4)	80 (2.6)	69 (2.2)	0.362
Blood transfusion	823 (13.1)	416 (13.3)	407 (13.0)	0.736
Sepsis	57 (0.9)	23 (0.7)	34 (1.1)	0.143
Hospitalization length				
Continuous (days)	9.93 ± 9.10	11.03 ± 9.68	8.82 ± 8.32	**<0.001**
≥7 days	2973 (47.4)	1724 (55.0)	1249 (39.8)	**<0.001**

Data are presented as number (percent) or mean ± standard deviation. Figures in bold denote statistical significance.

**Table 6:** Multivariable Cox proportional hazard model for the outcome of all-cause mortality at 1 year within the propensity score-matched sub-cohort.

	Univariable		Multivariable	
	HR (95% CI)	*p*-Value	AdjHR (95% CI)	*p*-Value
**Year of admission** **(continuous, per 1-year increase)**	0.98 (0.97–0.99)	**<0.001**	1.01 (1.00–1.02)	0.214
**Demographic details**				
Age ≥ 65 vs. <65 years	3.83 (3.01–4.87)	**<0.001**	2.27 (1.78–2.90)	**<0.001**
Sex male	0.68 (0.61–0.77)	**<0.001**	0.97 (0.86–1.10)	0.633
**Atherosclerotic** **cardiovascular risk factors**				
Diabetes mellitus	1.21 (1.08–1.36)	**0.001**	1.05 (0.93–1.19)	0.408
Dyslipidemia	0.58 (0.51–0.65)	**<0.001**	0.76 (0.67–0.86)	**<0.001**
Obesity	0.53 (0.44–0.64)	**<0.001**	0.78 (0.64–0.94)	**0.010**
**Cardiovascular** **comorbidities**				
Chronic coronary syndrome	0.44 (0.39–0.49)	**<0.001**	0.84 (0.73–0.96)	**0.009**
Peripheral arterial disease	1.96 (1.70–2.26)	**<0.001**	1.45 (1.25–1.68)	**<0.001**
Atrial fibrillation/flutter	1.86 (1.64–2.10)	**<0.001**	1.20 (1.06–1.37)	**0.005**
Clinical heart failure	2.06 (1.83–2.34)	**<0.001**	1.28 (1.13–1.46)	**<0.001**
**Non-cardiovascular** **comorbidities**				
Chronic obstructive pulmonary disease	2.14 (1.84–2.5)	**<0.001**	1.57 (1.34–1.85)	**<0.001**
Stage ≥ III chronic kidney disease	2.37 (2.11–2.67)	**<0.001**	1.33 (1.17–1.51)	**<0.001**
Anemia	2.40 (2.09–2.75)	**<0.001**	1.46 (1.27–1.68)	**<0.001**
Neurological disorders	2.39 (2.12–2.69)	**<0.001**	1.62 (1.44–1.83)	**<0.001**
Malignancy	3.04 (2.54–3.63)	**<0.001**	2.19 (1.83–2.62)	**<0.001**
Psychotic disorders	1.72 (1.27–2.32)	**<0.001**	1.39 (1.03–1.88)	**0.033**
**Clinical presentation**				
ST elevation (vs. non-ST elevation) myocardial infarction	0.57 (0.50–0.65)	**<0.001**	1.04 (0.90–1.20)	0.619
Right heart failure	2.08 (1.78–2.44)	**<0.001**	1.35 (1.15–1.60)	**<0.001**
**Hospital course**				
Invasive revascularization vs. conservative approach	0.21 (0.18–0.24)	**<0.001**	0.42 (0.35–0.51)	**<0.001**
Mechanical ventilation	2.34 (1.87–2.93)	**<0.001**	1.44 (1.14–1.82)	**0.002**
**Transthoracic** **echocardiography use**	0.65 (0.58–0.73)	**<0.001**	0.77 (0.68–0.88)	**<0.001**

Figures in bold denote statistical significance. AdjHR = adjusted hazard ratio; CI = confidence interval; HR = hazard ratio.

## Data Availability

The data underlying this article will be shared upon reasonable request to the corresponding author.

## Supplementary Materials

The following supporting information can be downloaded at https://www.mdpi.com/article/10.3390/jcdd13070322/s1. Figure S1: Love plot for balance in propensity score variables.

## Abbreviations

The following abbreviations are used in this manuscript:

AMI	Acute myocardial infarction
TTE	Transthoracic echocardiography
