# Supplementary figures and images for: Prevalence, Correlates, and Prognostic Significance of In-Hospital Transthoracic Echocardiography Use in Stable Acute Myocardial Infarction
Source: J Cardiovasc Dev Dis. 2026 Jul 10;13(7):322. doi: 10.3390/jcdd13070322 (PMC13411082; doi:10.3390/jcdd13070322)

## SUPPLEMENT

**Figure S1.** Love plot for balance in propensity score variables

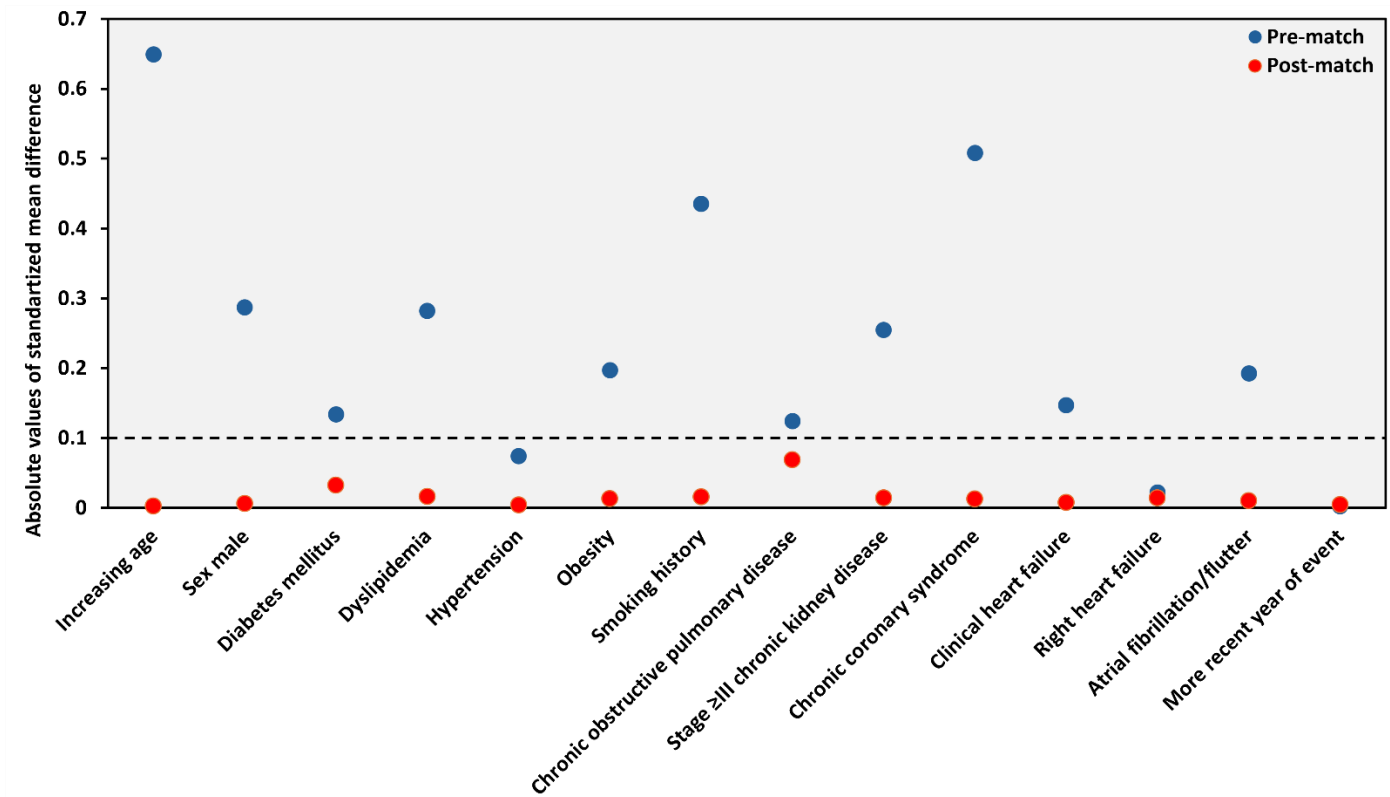

Supplement: Supplementary file 1 [file jcdd-13-00322-s001.zip › jcdd-4367201-supplementary.pdf]
